# Impact of Season, Demographic and Environmental Factors on *Salmonella* Occurrence in Raccoons (*Procyon lotor*) from Swine Farms and Conservation Areas in Southern Ontario

**DOI:** 10.1371/journal.pone.0161497

**Published:** 2016-09-09

**Authors:** Kristin J. Bondo, David L. Pearl, Nicol Janecko, Patrick Boerlin, Richard J. Reid-Smith, Jane Parmley, Claire M. Jardine

**Affiliations:** 1 Department of Pathobiology, University of Guelph, Guelph, Ontario, Canada; 2 Department of Population Medicine, University of Guelph, Guelph, Ontario, Canada; 3 Centre for Foodborne, Environmental and Zoonotic Infectious Diseases, Public Health Agency of Canada, Guelph, Ontario, Canada; 4 Department of Biology and Wildlife Diseases, University of Veterinary and Pharmaceutical Sciences Brno, Brno, Czech Republic; 5 Canadian Wildlife Health Cooperative, Department of Pathobiology, University of Guelph, Guelph, Ontario, Canada; Ross University School of Veterinary Medicine, SAINT KITTS AND NEVIS

## Abstract

*Salmonella* has been detected in the feces of many wildlife species, including raccoons (*Procyon lotor*), but little is known about the epidemiology of *Salmonella* in wildlife living in different habitat types. Our objective was to investigate demographic, temporal, and climatic factors associated with the carriage of *Salmonella* in raccoons and their environment on swine farms and conservation areas. Using a repeated cross-sectional study design, we collected fecal samples from raccoons and environmental samples (soil, manure pits, dumpsters) on 5 swine farms and 5 conservation areas in Ontario, Canada once every five weeks from May to November, 2011–2013. *Salmonella* was detected in 26% (279/1093; 95% CI 22.9–28.2) of raccoon fecal samples, 6% (88/1609; 95% CI 4.5–6.8) of soil samples, 30% (21/69; 95% CI 20.0–42.7) of manure pit samples, and 23% (7/31; 95% CI 9.6–41.0) of dumpster samples. Of samples testing positive for *Salmonella*, antimicrobial resistance was detected in 5% (14/279; 95% CI 2.8–8.3) of raccoon fecal, 8% (7/89; 95% CI 3.2–15.5) of soil, 10% (2/21; 95% CI 1.2–30.4) of manure pit, and 0/7 dumpster samples. Using multi-level multivariable logistic regression analyses, we found location type (swine farm or conservation area) was not a significant explanatory variable for *Salmonella* occurrence in raccoon feces or soil (p > 0.05). However, detection of *Salmonella* in raccoon feces was associated with rainfall, season, and sex with various interaction effects among these variables. We detected a variety of *Salmonella* serovars that infect humans and livestock in the feces of raccoons indicating that raccoons living near humans, regardless of location type, may play a role in the epidemiology of salmonellosis in livestock and humans in southwestern Ontario.

## Introduction

*Salmonella enterica* is one of the most important foodborne pathogens in the world [1] and is the 3rd most important cause of bacterial foodborne illness in Canada [2]. Although most non-typhoidal *Salmonella* infections are self-limiting and typically consist of uncomplicated gastroenteritis [3], some infections can require antimicrobial therapy, including invasive infections and infections among children, seniors and immunocompromised patients [4]. Antimicrobial resistant *Salmonella* infections in humans have been associated with increased risk of extraintestinal infections, hospitalization, longer duration of illness [5, 6], and an excess number of cases [7], compared to susceptible isolates.

Transmission of *Salmonella* from animals to humans typically occurs via the fecal-oral route through consumption of contaminated food products [8, 9], water [10–12], or direct contact with animals [13]. Although the intestinal tracts of production animals are considered to be the primary reservoir for non-typhoidal *Salmonella* [14], these bacteria are also found in many wildlife species [15]. Wildlife may ingest *Salmonella* through consumption of contaminated animal feed or water, or through contact with contaminated farm buildings, manure, or direct contact with other animals carrying the bacteria [15]. *Salmonella* can persist in water, soil, and on surfaces, and can survive for at least a year in soil [16], from weeks to months in water and on plants [17], and up to a month in infected pig waste slurry after it is spread into the environment [18]. *Salmonella* can enter aquatic systems through treated and untreated sewage [19–20] and urban and agricultural run-off [21], which might increase the risk of exposure to wildlife living near these areas. *Salmonella* diversity and abundance in water samples is strongly influenced by seasonal precipitation and water temperature [22], and detection in soil samples is associated with moisture [23], and human and animal activity [18].

Proximity to swine farms is associated with an increase in the likelihood of *Salmonella* occurrence in wild bird species living at high densities [24], and *Salmonella* is detected more frequently in fecal swabs and environmental samples (e.g., livestock feed, litter, and soil) from swine farms than poultry and dairy farms, suggesting that swine farm environments might be important reservoirs of *Salmonella* [25]. *Salmonella* is also more prevalent in wildlife living close to human habitation [26–28]. Recently, Miller et al. [29] found that the probability of *Salmonella* carriage in wild mongooses in the West Indies increased with higher human density and decreased distance from roads. Similarly, the prevalence of antimicrobial resistant bacteria has been found more frequently in small rodents living in close proximity to humans and agriculture than in areas with little anthropogenic influence [30–32], suggesting local contamination of the environment as a potential source. Wildlife living in areas used for agriculture or inhabited by humans may transmit *Salmonella* [15, 33] and resistant bacteria they are carrying in their feces to livestock and to humans [34]. Identifying the pathways that allow the transmission of *Salmonella* and resistant bacteria at the human-wildlife interface will increase our ability to manage this foodborne pathogen.

According to previous studies, 15–27% of apparently healthy raccoons in North America shed *Salmonella* in their feces [35, 36], suggesting that raccoons may play a role in the epidemiology of *Salmonella*, which infects humans and livestock, and contaminates the environment. Raccoons can achieve high population densities of 37–94 raccoons/km^2^ in urban areas [37], inhabit both urban and rural habitats, can move between these location types, and have relatively large home ranges, up to 4 km^2^ in Ontario [38]. Although *Salmonella* has been isolated and reported from a wide variety of wildlife, there have been few multi-year studies of *Salmonella* in free-ranging wild animals [26, 28]. Using a repeated cross-sectional study conducted over 3 years, our objectives were to: 1) compare *Salmonella* prevalence, *Salmonella* serovars, and antimicrobial resistance patterns detected in *Salmonella* from raccoon and environmental samples on swine farms and conservation areas; and 2) assess the impact of seasonal, climatic, annual, location, and raccoon demographic factors on the occurrence of *Salmonella* in raccoon fecal and environmental samples.

## Materials and Methods

**Fig 1:**
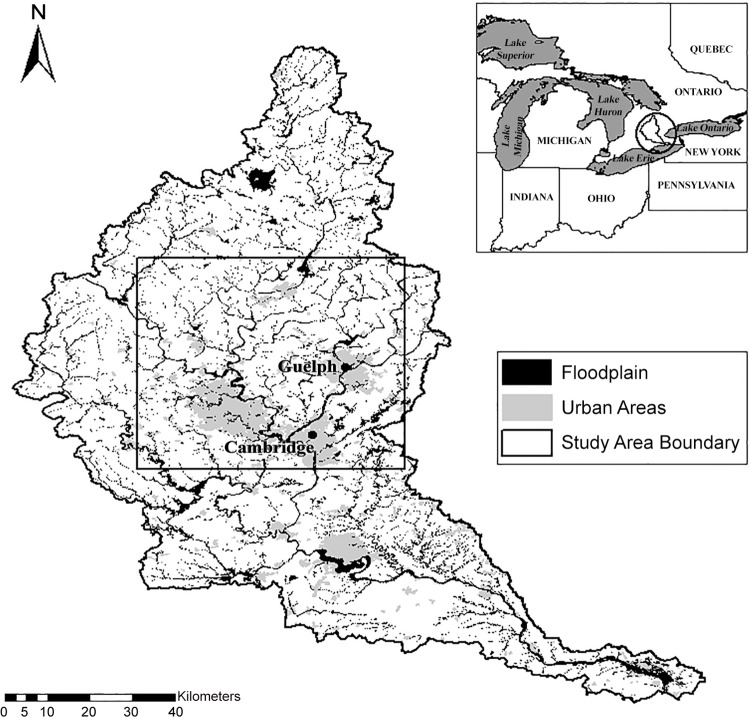
Map of the study area and Grand River Watershed in southern Ontario, Canada. Study Area [map]. Data layers: Grand River Conservation Authority: floodplain, watershed boundary; Open Government License–Ontario: built up areas; Great Lakes Commission: Great Lakes States boundaries, main lakes, province boundaries [computer files]. University of Guelph, Guelph, ON: Generated by Kristin Bondo, August 21, 2015. Using: ArcMap [GIS]. Version 10.1, Redlands, CA: Esri, 2012.

Procedures for trapping and handling raccoons were approved by the Animal Care Committee at the University of Guelph following the guidelines of the Canadian Committee on Animal Care. Raccoons were live-trapped on 5 swine farms and 5 conservation areas from May through November, 2011**–**2013. All sites were located within the boundaries of the Grand River watershed in Ontario within a 100-km radius of either Guelph or Cambridge (Fig 1). Distance between sites ranged from 1.3 to 52.2 km. The Grand River watershed is approximately 6800 km^2^ and is the largest watershed in Ontario. Predominant land use in the watershed is agricultural (75%). Of the agricultural land, 50% contains crops and the rest livestock. The watershed is also heavily impacted by a large urban population (>600,000) concentrated in the central portion. Thirty waste water treatment plants discharge into the Grand River and its tributaries [39]. Each site was trapped once every 5 weeks during this period, and environmental samples were collected at each site during the same week that animals were trapped.

All of the farm sites selected for this study were part of FoodNet Canada, a sentinel site based enteric pathogen surveillance program [40], and identified themselves as being primarily swine farms. The farms were chosen based on their proximity to the University of Guelph, and included farrow-to-young grower and farrow-to-finish operations. Up to 2500 pigs were housed indoors on each farm. In addition to pigs, one farm had 100**–**110 dairy cattle on site, another farm had approximately 100 layer chickens housed indoors, and another swine farm had a poultry operation and horses and ponies on site. Only waste from pigs was stored in the manure pits sampled in this study and poultry manure was not spread on the farms’ fields. Three of the five farms did not administer injectable antimicrobials or antimicrobials in-feed to swine. The other two farms administered both injectable and in-feed antimicrobials to swine.

The conservation areas ranged in size from 75**–**1608 ha and were all located primarily in peri-urban areas. Habitat types within the conservation areas included mixed hardwood and coniferous forests, ponds, lakes, and wetlands. Recreational activities in many of these areas included hiking, fishing, picnicking, camping, and swimming.

### Sample Collection

Raccoons were live-trapped and processed as described previously [41]. Briefly, at each site, 20**–**40 Tomahawk live traps (Tomahawk Live Trap Co.; Tomahawk, Wisconsin, USA) were set 3–4 nights/week at each site in areas with limited public access, but where raccoons were known to be present, including around dumpsters and buildings. Upon capture, raccoons were anesthetized using an intramuscular injection of 0.025 mg/kg dexmedetomidine hydrochloride (Dexdomitor 0.5 mg/ml; Pfizer Animal Health, Kirkland, Quebec, Canada) and 5 mg/kg ketamine hydrochloride (Vetalar 100 mg/ml; Bioniche Animal Health, Belleville, ON, Canada). For subsequent identification, a numbered metal ear tag (1005–3, National Band and Tag Co.; Newport, Kentucky, USA) was placed in one ear and a passive integrated transponder tag (GPT12 Pre-Load Sterile; Biomark, Boise, Idaho, USA) was injected subcutaneously between the shoulder blades. Sex, age class (adult or juvenile, on the basis of animal size and teeth wear/staining), and body mass were recorded for each animal. Fecal swabs were collected per rectum using Cary-Blair applicators (BBL CultureSwab, BD; Becton, Dickinson and Company, Annapolis, Maryland, USA). Although individuals were only sampled once per trapping week, multiple samples were collected from the same individual if they were caught in subsequent trapping sessions.

Ten to twenty soil samples were collected within a 2-m radius of the traps on the first day of each trapping week at each study site. Approximately 10 g of soil, free of obvious fecal contamination, was collected into sterile containers. At each swine farm, one manure pit sample was collected on the first day of each trapping week. To collect manure pit samples, a 24’ Nasco Swing Sampler (Conbar, Monroeville, NJ, USA) was used to collect three sub-samples from three locations around the pit, and up to two depths (i.e., the top 1/3, and mid depth of the storage), for a total of 6 sub-samples. The sub-samples were poured into a sampling bucket with a clean plastic liner and mixed to create the pooled sample and then transferred into a sterile vial. During 2013, dumpster samples were collected, when available, each month, from 2**–**3 dumpsters/site from three conservation areas. The other two conservation areas were not sampled because dumpsters were not present on these sites. Dumpster samples were collected < 1 day after dumpsters were emptied by swabbing the bottom of the dumpster using a Swiffer® wipe (Armstrong, Proctor and Gamble, Cincinnati, Ohio, USA) attached to a 1**–**2 m extension pole (Bennett, Concord, Ontario, Canada). Swiffer® wipes were kept moist by placing them in Whirl-pak bags (Nasco, Fort Atkinson, Wisconsin, United States) containing approximately 20 ml of sterile 0.85% saline solution kept at 4°C before and after sampling.

### Laboratory Work

All sample types were labeled and placed in a cooler in the field and refrigerated upon return to the laboratory before being submitted for *Salmonella* isolation. The methods used have been described previously [35]. To prepare the dumpster samples for assay, 50 ml of buffered peptone water was added to the Swiffer® sample, mixed and incubated at 37°C for 24hrs. Then 0.1 ml of liquid was inoculated into modified semi-solid *Rappaport vassiliadis* and incubated at 42°C for 24–72hrs.

*Salmonella* isolation was performed at the McEwen Lab, Canadian Research Institute for Food Safety, University of Guelph. One *Salmonella* isolate from each positive sample was submitted for serotyping, phagetyping (for serotypes Typhimurium, Enteritidis, and Heidelberg), and antimicrobial susceptibility testing at the National Microbiology Laboratory (formerly the Laboratory for Foodborne Zoonoses), Public Health Agency of Canada (Guelph, Ontario, Canada).

The National Antimicrobial Monitoring System (NARMS) CMVA2GNF plate was used for antimicrobial susceptibility testing; it includes 15 antimicrobials: amoxicillin-clavulanic acid (AMC), ampicillin (AMP), azithromycin (AZM), cefoxitin (FOX), ceftiofur (TIO), ceftriaxone (CRO), chloramphenicol (CHL), ciprofloxacin (CIP), gentamicin (GEN), kanamycin (KAN), nalidixic acid (NAL), streptomycin (STR), sulfisoxazole (SOX), tetracycline (TCY), and trimethoprim-sulfamethoxazole (SXT). Minimum inhibitory concentration breakpoints were those used by the Canadian Integrated Program for Antimicrobial Resistance Surveillance (CIPARS) and NARMS [42]. Based on these breakpoints, isolates were classified as susceptible, intermediate, or resistant. For this study, we considered all isolates classified as intermediate or resistant to be resistant.

### Statistical Modeling

The primary outcome variable for raccoon fecal, soil, manure pit, and dumpster samples was *Salmonella* (positive vs. negative). To compare the prevalence of *Salmonella* in raccoon fecal, soil, manure pit, and dumpster samples, type of sample was included as a fixed effect. For all other statistical modeling, samples were analyzed by sample type. Explanatory variables included, if applicable, raccoon sex (male or female), raccoon age (adult or juvenile), location type (swine farm or conservation area), year (2011**–**2013), sum of rainfall over 30 days prior to sample collection, mean temperature over 30 days prior to sample collection, and season (May–July or August–November). Two distinct seasons were considered: rearing (May–July) and pre-denning/dispersal (August–October) as defined by Rosatte et al. [38]. Sum of rainfall and mean temperature prior to 30 days of sample collection were used because this time period for rainfall was found to be significantly associated with *Salmonella* carriage in free-ranging wildlife in other studies [29, 43]. Mean daily temperature and total rainfall per day were downloaded from Environment Canada from the nearest weather station with complete data (Fergus Ministry of the Environment (MOE), ON) from 2011 to 2013. Missing values were filled in using data from the next nearest weather station in Guelph, Ontario.

Raccoon fecal and soil samples were modelled using multi-level univariable and multi-variable logistic regression. Univariable models were initially constructed and then variables were retained in the final model if they were significant, part of a significant interaction term or acted as a confounding variable. A variable was considered to be a confounding variable if it was a non-intervening variable and its removal from the model resulted in ≥ 20% change in the coefficients of any statistically significant variable [44]. The significance level was set at α = 0.05. Additional details regarding the building of multi-variable models are described in the S1 Appendix.

Manure pit and dumpster samples were analyzed using univariable exact logistic regression due to a small effective sample size for these dependent variables. No statistical tests were conducted on antimicrobial resistant results due to the rarity of this outcome being detected. To determine the association between the presence of the most common *Salmonella* serovars in raccoon feces (Oranienburg, Newport, I:4,[5],12:b:-, and Thompson analyzed separately) in relation to all other *Salmonella* serovars, univariable logistic regression models with random effects were also used. Factors examined for association with the most common *Salmonella* serovars included year, location type, season, age, and sex of the raccoon.

For each model, the odds ratio and 95% confidence interval of each variable were reported. The significance of main effects, categorical variables with more than two categories and interaction terms were assessed using Wald’s χ^2^ test. Independent continuous variables that had nonlinear relationships with the outcome variable based on a significant quadratic term and on visual assessment of the lowess curve were categorized or modelled as a quadratic relationship if appropriate.

Random effects were included to account for autocorrelation among samples taken from the same site, animal, manure pit, or dumpster. A random effect was excluded from the final model if the variance components were very small and if the model fit was not improved based on assessing changes in Akaike’s Information Criterion (AIC) and Bayesian Information Criterion (BIC) values to the removal of the random effect. If the model fit was not improved by the inclusion of random effects, then ordinary logistic regression was used to analyze the data. To determine the amount of variation explained by each organizational level (i.e., site and sample levels), variance partition coefficients (VPCs) were estimated from the variance components of the final multi-level logistic regression models using the latent variable technique [44]. All statistical tests were conducted using STATA (STATA Intercooled 13.0; StataCorp, College Station, Texas, USA).

### Assessing model fit

For multi-level models, Pearson and deviance residuals were used to determine if there were any outlying observations, and best linear unbiased predictors (BLUPs) of the random effects were examined to assess overall model fit. Specifically, we graphically assessed if the BLUPs fulfilled the assumptions of normality and homogeneity of variance. If the BLUPS did not meet these model assumptions, we compared models with and without the random intercept(s) using AIC and BIC to confirm that the addition of these terms improved model fit.

For ordinary logistic regression models with continuous independent variables, the Hosmer-Lemeshow goodness-of-fit test was used to assess model fit and for those without continuous variables, Pearson’s goodness-of-fit test was used. In addition, for regular logistic regression models, leverage, delta-beta, Pearson, standardized Pearson and deviance residuals were used to visually assess if there were any outlying observations for these models. If any outliers were found in either the multi-level or regular logistic regression models, they were investigated for recording errors; otherwise, they were left in the model.

## Results

We collected 1096 fecal samples from 627 individual raccoons, 1609 soil samples, 69 manure pit samples, and 31 dumpster samples. Three individual raccoons were caught at two different swine farms during the study, so we randomly selected and excluded one result each from the analyses. Sex and age were undetermined for one and three raccoons, respectively. *Salmonella* was detected in 26% raccoon fecal, 6% soil, 30% manure pit, and 23% dumpster samples (Table 1). The proportion of all sample types testing positive for *Salmonella* by age, sex, location type, season, and year categories are also presented in Table 1.

**Table 1:** Proportion (%) of raccoon fecal, soil, manure pit, and dumpster samples testing positive for *Salmonella* overall and by age, sex, location type, season, and year in Ontario from May–November 2011–2013.^a^

		Feces	Soil	Manure Pit	Dumpster
Predictor	Category	% Positive (95% CI)	% Positive (95% CI)	% Positive (95% CI)	% Positive (95% CI)
		(*n* = 1093) ^c^	(*n* = 1609) ^c^	(*n* = 69) ^c^	(*n* = 31) ^c^
Age ^b^	Adult	23.5 (20.5**–**26.6)	—	—	—
	Juvenile	30.0 (25.0–34.9)	—	—	—
Sex ^b^	Female	22.8 (19.4 26.3)	—	—	—
	Male	28.4 (24.5–32.3)	—	—	—
Location type	Swine farm	22.8 (18.7–26.9)	5.7 (4.2–7.5)	30.4 (20.0–42.7)	—
	Conservation	27.1 (23.8–30.5)	5.4 (3.9–7.1)	—	22.6 (9.6–41.0)
Season	May to July	22.2 (18.9–25.6)	3.0 (1.9–4.5)	17.2 (2.6–31.9)	38.5 (13.9–68.4)
	Aug. to Nov.	29.4 (25.4–33.4)	7.5 (5.8–9.4)	40.0 (24.1–55.9)	11.1 (1.4–34.7)
Year	2011	29.0 (24.2–33.8)	6.3 (4.5–8.6)	55.0 (31.1–78.9)	—
	2012	26.3 (22.2–30.4)	7.2 (5.1–9.8)	33.3 (13.1–53.7)	—
	2013	20.3 (15.8–24.9)	2.9 (1.7–4.8)	8.0 (1.0–26.0)	22.6 (9.6–41.0)
**Overall**		**25.5 (22.9–28.2)**	**5.5 (4.5–6.8)**	**30.4 (20.0–42.7)**	**22.6 (9.6–41.0)**

^a^ Dash indicates “Not Applicable”.

^b^Age was unknown for 3 raccoons and sex was unknown for 1 raccoon.

^c^ n = total number of samples.

### Comparison of Sample Types

*Salmonella* was detected more frequently in raccoon feces (OR = 6.71; 95% CI, 4.89–9.25; p ≤ 0.001), manure pits (OR = 9.64; 95% CI, 4.94–18.78; p ≤ 0.001), and dumpsters (OR = 5.7; 95% CI, 2.10–15.60; p ≤ 0.001) than in soil. There were no significant differences between *Salmonella* presence in raccoon fecal (OR = 0.70; 95% CI, 0.37–1.31; p = 0.264) and dumpster samples (OR = 0.59; 95% CI, 0.19–1.88; p = 0.375) compared to manure pit samples or between raccoon fecal and dumpster samples (OR = 1.17; 95% CI, 0.44–3.12; p = 0.750). Based on the VPC estimates of this model, site, individual animal/manure pit/dumpster, and sample levels explained 1.4%, 18.9%, and 79.7%, respectively, of the variance in *Salmonella* occurrence.

### Raccoon Fecal Samples

In the univariable models, *Salmonella* occurrence in raccoon feces was significantly associated with sex, season and rainfall (S1 Table). The final multivariable model for raccoon fecal samples included sex, season, rainfall and season-sex and season-rainfall interaction terms (Table 2).

**Table 2:** Results from multi-level multivariable and exact ^a^ logistic regression models showing associations between the occurrence of *Salmonella* in raccoon fecal and soil samples with respect to raccoon sex for raccoon samples, year, season, rainfall and interaction effects in Ontario, Canada.

		Multivariable models for *Salmonella* according to sample type								
Predictor	Sub-Category	Raccoon feces ^b^			Soil ^b^^,^ ^c^			Manure Pit ^b^^,^ ^d^		
		(*n* = 1092)			(*n* = 1609)			(*n* = 69)		
		Odds Ratio	95% CI	*P*	Odds Ratio	95% CI	*P*	Odds Ratio	95% CI	*P*
Sex	Female	REF			—	—	—	—	—	—
	Male	**2.06**	**1.28–3.32**	**0.003**	—	—	—	—	—	—
Season	May to July	REF			REF			REF		
	Aug. to Nov.	**5.27**	**2.18–12.77**	**< 0.001**	**2.74**	**1.66–4.54**	**< 0.001**	**3.86**	**1.00–17.68**	**0.044**
Year ^e^	2012 (2011 REF)	—	—	—	1.26	0.78**–**2.04	0.335	0.42	0.09–1.75	0.210
	2013 (2011 REF)	—	—	—	**0.45**	**0.24–0.83**	**0.011**	**0.06**	**0.006–0.42**	**< 0.001**
Rainfall	Sum prior 30 days	1.00	0.99**–**1.00	0.155	—	—	—	—	—	—
Interactions ^f^	Sex*Season	**0.50**	**0.26–0.94**	**0.031**	—	—	—	—	—	—
	Rainfall*Season	**0.99**	**0.98–1.00**	**0.014**	—	—	—	—	—	—
Variance [VPC]	Site-level	0.12 [2.8]	0.02–0.58		—	—	—	—	—	—
	Animal-level	0.79 [18.7]	0.32–1.93		—	—	—	—	—	—
	Sample-level	[78.5]	—		—	—	—	—	—	—

^a^ Exact logistic regression was used to model manure pit samples.

^b^ Random effects included site and animal. Significant differences are in bold, *n* = total number of samples, the dash indicates “Not Applicable”, REF = referent group, and CI = confidence interval.

^c^ Random effect for site was not included in the model because it did not improve model fit based on AIC and BIC; it explained only a small amount of the variance (0.023), and its removal had little to no impact on the coefficients in the model.

^d^ Interactions were not tested for manure pit samples and random effects were not included in the model.

^e^ Wald’s χ^2^ test for year was *P* = 0.004 for soil samples. Results for 2013 versus 2012 for soil samples and manure pit samples were (OR = 0.39; 95% CI, 0.21–0.72; p = 0.003) and (OR = 0.16; 95% CI, 0.01–0.97; p = 0.029), respectively.

^f^ To interpret season, sex, rainfall, and their interaction effects, refer to contrasts in Table 3.

The contrasts derived from the models showed that male raccoons were more likely to carry *Salmonella* during May to July than female raccoons (Table 3). In addition, male and female raccoons were more likely to carry *Salmonella* during August to November than May to July (Table 3).

**Table 3:** Contrasts derived from the multi-level logistic regression model for the presence of *Salmonella* in raccoon feces (Table 2) from Ontario, Canada to interpret interaction effects between raccoon sex and season.

	Contrast	Sub-Category	Odds Ratio	95% CI	*P*
1.	Male vs. Female (REF) ^a^^,^ ^b^	May–July	**2.06**	**1.28–3.32**	**0.003**
		Aug.–Nov.	1.02	0.64–1.63	0.953
2.	Aug.–Nov. vs. May–July (REF) ^a^^,^ ^c^	Male	**2.16**	**1.10–6.14**	**0.031**
		Female	**5.22**	**2.17–12.55**	**< 0.001**

^a^ Significant differences are in bold, REF = referent group, and CI = confidence interval.

^b^ A contrast examining the relationship between *Salmonella* carriage between males and females during different seasons.

^c^ A contrast examining the relationship between *Salmonella* carriage between seasons for males and females.

**Fig 2:**
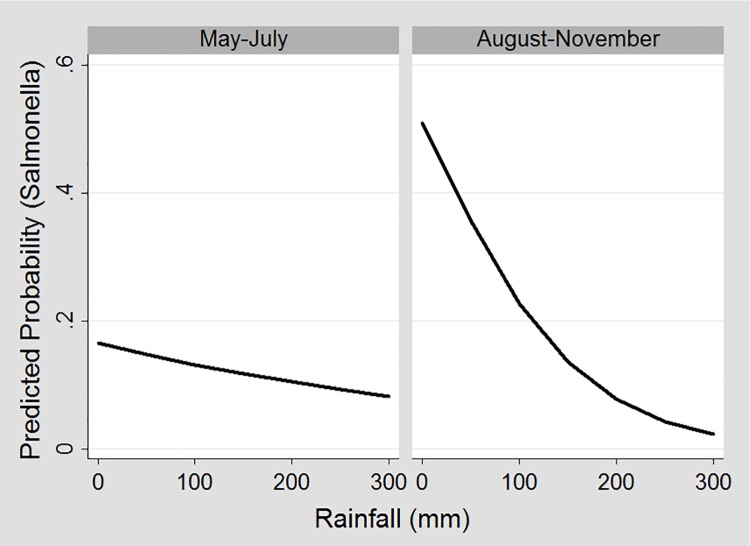
Predicted probability of fecal samples testing positive for *Salmonella* during different seasons at different levels of rainfall over 30 days prior to sample collection. ^a a^ The graph for female raccoons is displayed.

Higher rainfall was associated with a lower predicted probability of *Salmonella* in raccoon feces in both seasons; however, the relationship was much more pronounced August to November than May to July (Fig 2).

### Environmental samples

In the univariable models, *Salmonella* detection in soil samples was significantly associated with season and year, and there was a significant quadratic relationship between *Salmonella* occurrence in soil samples and mean temperature over 30 days prior to sample collection (S1 Table). The final multivariable model for *Salmonella* occurrence in soil samples included year and season. In this model, soil samples collected between August and November were more likely to be *Salmonella* positive than samples collected between May to July (Table 2). In addition, soil samples collected in 2013 were less likely to be *Salmonella* positive than samples collected in 2011 and 2012 (Table 2).

Manure pit samples were significantly less likely to be *Salmonella* positive in 2013 than in 2011 and 2012 in the univariable analysis (S1 Table). In the main effects model for manure pit samples, *Salmonella* was significantly more likely to occur in samples collected in August to November than May to July and less likely to occur in year 2013 than in 2011 and 2012 (Table 2). In the univariable analysis for dumpster samples for season, there was no significant difference in *Salmonella* occurrence in dumpster samples August to November compared to May to July (OR = 0.2; 95% CI, 0.02–1.64, p = 0.099).

### *Salmonella* serovars

*Salmonella* serovars detected in raccoon fecal, soil, manure pit, and dumpster samples are presented by sample type and location type (Table 4).

**Table 4:** Percentage (95% CI) of raccoon fecal, soil, manure pit, and dumpster samples testing positive for *Salmonella* that were detected and serotyped for each serovar overall and according to location type in southwestern Ontario from May–November 2011–2013.^a^

					Conservation Area		Swine Farm	
Serovar	Feces	Soil	Manure pit	Dumpster	Feces	Soil	Feces	Soil
	(*n* = 279)	(*n* = 89)	(*n* = 21)	(*n* = 7)	(*n* = 187)	(*n* = 42)	(*n* = 92)	(*n* = 47)
Newport	21.5	18.0	—	42.8	14.4	7.1	35.9	27.6
	(16.8**–**26.8)	(10.6–27.5)		(9.9–81.6)	(9.7–20.3)	(1.5–19.5)	(26.1–46.5)	
Oranienburg	16.1	9.0	—	—	23.5	19.0	1.1	—
	(12.0**–**21.0)	(4.0–16.9)			(17.6–30.3)	(8.6–34.1)	(0.03–5.9)	
I:4,[5],12:b:-	13.6	6.7	—	—	15.5	9.5	9.8	2.6
	(9.8**–**18.2**)**	(2.5**–**14.1)			(10.6–21.5)	(2.6–22.6)	(4.6–17.8)	(0.3–9.1)
Thompson	12.9	19.1	—	14.3	16.6	28.6	5.4	10.6
	(9.2**–**17.4)	(11.5–28.8)		(0.4–57.9)	(11.6–22.7)	(15.7–44.6)	(1.8–12.2)	(3.5–23.1)
Typhimurium	10.4	13.5	4.8	14.3	11.8	14.3	7.6	12.8
	(7.1**–**14.6)	(7.2–22.4)	(0.1–23.8)	(0.4–57.9)	(7.5–17.3)	(54.2–28.5)	(3.1–15.0)	(4.8–25.7)
Agona	5.7	9.0	23.8	—	—	—	17.4	17.0
	(3.3**–**9.1)	(4.0–16.9)	(8.2–47.2)				(10.3–26.7)	(7.6–30.8)
Infantis	5.0	5.6	14.3	—	3.2	—	8.7	10.6
	(2.8**–**8.3)	(1.8–12.6)	(3.0–36.3)		(1.2–6.9)		(3.8–16.4)	(3.5–23.1)
Heidelberg	3.2	2.2	—	—	4.3	4.8	1.1	—
	(1.5**–**6.0)	(0.3–7.9)			(1.9–8.3)	(0.6–16.2)	(0.03–5.9)	
Hartford	1.4	2.2	—	—	0.5	2.4	3.3	2.1
	(0.4**–**3.6)	(0.3–7.9)			(0.01–2.9)	(0.01–12.6)	(0.7–9.2)	(0.05–11.3)
Johannesburg	1.1	—	—	—	1.6	—	—	—
	(0.2**–**3.1)				(0.3–4.6)			
Litchfield	1.1	—	—	—	1.6	—	—	—
	(0.2**–**3.1)				(0.3–4.6)			
Berta	0.7	—	—	—	1.1	—	—	—
	(0.01–2.6)				(0.1–3.8)			
Enteritidis	0.7	1.1	—	—	0.5	—	1.1	2.1
	(0.01–2.6)	(0.03–6.1)			(0.01–2.9)		(0.03–5.9)	(0.05–11.3)
Hadar	0.7	1.1	—	—	0.5	—	1.1	2.1
	(0.01–2.6)	(0.03–6.1)			(0.01–2.9)		(0.03–5.9)	(0.05–11.3)
I:Rough-O:b:-	0.4	1.1	—	—	0.5	2.4	—	—
	(0.001–2.0)	(0.03–6.1)			(0.01–2.9)	(0.01–12.6)		
Give	0.4	2.2	—	—	0.5	4.8	—	—
	(0.001–2.0)	(0.3–7.9)			(0.01–2.9)	(0.6–16.2)		
IIIb:11:k:z53	0.4	1.1	—	—	—	—	1.1	2.1
	(0.001–2.0)	(0.03–6.1)					(0.03–5.9)	(0.05–11.3)
Poona	0.4	2.2	4.8	—	—	—	1.1	4.2
	(0.001–2.0)	(0.3–7.9)	(0.1–23.8)				(0.03–5.9)	(5.2–14.5)
Schwarzengrund	0.4	—	—	14.3	—	—	1.1	—
	(0.001–2.0)			(0.4–57.9)			(0.03–5.9)	
Livingstone	—	1.1	9.5	—	—	—	—	2.1
		(0.03–6.1)	(1.2–30.4)					(0.05–11.3)
Livingstone var. 14+	—	—	33.3	—	—	—	—	—
			(14.6–57.0)					
Other serovars^b^	3.9	4.5	9.5	14.3	3.7	7.1	4.3	2.1
	(2.0–6.9)	(1.2–11.1)	(1.2–30.4)	(0.4–57.9)	(1.5–7.6)	(1.5–19.5)	(1.2–10.8)	(0.05–11.3)
**Total *Salmonella* Positive**	**25.5**	**5.5**	**30.4**	**22.6**	**27.1**	**5.2**	**22.8**	**5.8**
**Total sample size**	**1093**	**1609**	**69**	**31**	**689**	**803**	**404**	**806**

^a^ The dash indicates none were detected; *n* = total number of samples testing positive for *Salmonella*.

^b^ Other serovars are those that were detected once and in only one sample type. They include the following:

• Fecal, conservation area: I:Rough-O:e,h:1,2, I:Rough-O:k:1,5, ssp. I:4,5,12:i:- (U291), ssp. I:6,8:e,h:-, Kentucky, Montevideo, Oranienburg var. 14+

• Soil, conservation area: IIIb:11:k:-, ssp. I:4,12:i:- (U291), ssp. I:11:k:-

• Dumpster: Pomona

• Fecal, swine farm: Infantis var 14+, Braenderup, Molade, Rissen

• Soil, swine farm: Kiambu

• Lagoon: I:Rough-O:l,v:e,n,z15, Mbandaka

Thirty and 19 *Salmonella* serovars were detected in raccoon feces and soil samples, respectively. *Salmonella* serovars Newport, Oranienburg, I,4,[5],12:b-, and Thompson were the most commonly detected in raccoon fecal samples, and Thompson, Newport, Typhimurium, Oranienburg and Agona were among the most common in soil samples (Table 4).

In the univariable analysis for the most common serovars detected in raccoon fecal samples, *Salmonella* Newport was more likely to occur in raccoon feces than other serovars on swine farms than conservation areas (S2 Table), and *Salmonella* Oranienburg was more likely to occur than other serovars in female than male raccoon feces, less likely for juveniles than adults, and more likely in 2012 than 2011 (S2 Table). There were no significant associations in any of the univariable analyses for *Salmonella* I, 4,[5],12:b- or Thompson (S2 Table).

### Diagnostics for Residual Analyses

There were no outlying observations with recording errors in any of the multi-level or logistic regression models. Although the BLUPS were not normally distributed in all of the multi-level models, the random effects were included in the models since model fit was improved based on the reduction in AIC and BIC when these effects were included. For models constructed using regular logistic regression (i.e., no random effects), all models fit the data based on non-significant Hosmer-Lemeshow (binary data) and Pearson goodness-of-fit (binomial data) tests.

### Antimicrobial Resistance

Among the samples that tested positive for *Salmonella*, antimicrobial resistance to one or more of the 15 antimicrobials tested was detected in 5% (14/279; 95% CI 2.8–8.3) of raccoon fecal, 8% (7/89; 95% CI 3.2–15.5) of soil, 10% (2/21; 95% CI 1.2–30.4) of manure pit, and 0/7 dumpster isolates (Table 5).

**Table 5:** Antimicrobial resistance patterns in *Salmonella* positive raccoon fecal, soil, and manure pit samples on five conservation areas (Site no: 1–5) and five swine farms (Site no: 5–10) in southwestern Ontario from May to November 2011–2013.

Location Type	Year	Site	*Salmonella*	Fecal	Soil	Manure pit	AMR Pattern ^b^^,^ ^c^
		No.	Serovar/(Phagetype) ^a^	(no.)	(no.)	(no.)	
**Conservation Area**	2011	1	Heidelberg (19)	4	—	N/A	AMP
		1	Heidelberg (19)	1	1	N/A	AMP–SXT
		1	Heidelberg (19)	—	1	N/A	AMP–STR–SXT
		1	Oranienburg	1	—	N/A	CHL ^c^
	2012	2	Hadar	1	—	N/A	STR–TCY
		1	Oranienburg	1	1	N/A	CHL ^**c**^
		1	Kentucky	1	—	N/A	STR–TCY
		1	I:4,12:i:- (U291)	—	1	N/A	AMP–CRO–TIO
	2013	3	Heidelberg (29)	1	—	N/A	AMC–AMP–CRO–FOX–TIO
**Swine Farm**	2011	8	I:4,12:b:-	1	—	—	FOX ^**c**^
	2012	9	Agona	—	1	1	CHL ^**c**^
	2013	9	Typhimurium var. Copenhagen (104)	—	—	1	SOX–STR–TCY
		7	Typhimurium var. Copenhagen (104)	1	—	—	SOX–STR–TCY
		6	Hadar	1	1	—	STR–TCY
		6	Kiambu	—	1	—	AMP–TCY
		6	Schwarzengrund	1	—	—	SOX–STR–TCY

^a^ The number of isolates detected for each serovar and phagetype are listed for each sample type where applicable.

The dash indicates none were detected. N/A = not applicable; no. = number.

^b^ AMC = amoxicillin-clavulanic acid; AMP = ampicillin; CHL = chloramphenicol; CRO = ceftriaxone; FOX = cefoxitin; SOX = sulfizoxazole; STR = streptomycin; SXT = trimethoprim sulfamethoxazole; TIO = ceftiofur; TCY = tetracycline.

^c^ AMR patterns had intermediate antimicrobial susceptibility, but were considered to be resistant in this study.

Of the *Salmonella* positive samples, antimicrobial resistance was detected in 5.2% (10/187; 95% CI 2.6–9.6) of raccoon fecal and 9.5% (4/42; 95% CI 2.6–22.6) of soil samples from conservation areas as compared to 4.3% (4/92; 95% CI 1.2–10.8) of raccoon fecal, 6.4% (3/47; 95% CI 1.3–17.5) of soil, and 9.5% (2/21; 95% CI 1.2–30.4) of manure pit samples from swine farms. Of swine farm samples testing positive for *Salmonella*, antimicrobial resistance was detected in 7.3% (3/41; 95% CI 1.5–19.9) of raccoon fecal and in 8.7% (2/23; 95% CI 1.1–28.0) of soil samples from swine farms that reported using antimicrobials as compared with 2.0% (1/51; 95% CI 0.05–10.4) of raccoon fecal and in 4.3% (1/23; 95% CI 0.1–21.9) of soil samples from farms that reported not using antimicrobials.

The most common resistance observed was to AMP (10/23), TCY (8/23) and STR (8/23); resistance to antimicrobials of very high importance [45] was observed in only 2/23 isolates and from only conservation areas (Table 5). Resistance to more than one antimicrobial was observed in 56% (13/23) of resistant isolates. Resistance to three or more classes of antimicrobials were detected in four *Salmonella* isolates of three serovars: Heidelberg (PT 19) from a soil sample from a conservation area, Typhimurium (DT104) from a raccoon fecal and manure pit sample from a swine farm, and Schwarzengrund from a raccoon fecal sample from a swine farm. Although all isolates with intermediate susceptibility were considered to be resistant for the purpose of this study, intermediate susceptibility was found in 26% (6/23) of the isolates, and in all occasions it was found to FOX or CHL (Table 5).

## Discussion

We predicted that raccoons and soil samples on swine farms would have greater exposure to environmental sources of *Salmonella* than raccoons living on conservation areas. Location type did not influence *Salmonella* occurrence in raccoon fecal or soil samples, which was in contrast to Pedersen et al. [46] who found higher *Salmonella* prevalence in Rock Pigeons (*Columba livia*) on dairy farms than urban areas and to Navarro-Gonzalez et al. [28] who found higher *Salmonella* prevalence in free-ranging wild boars (*Sus scrofa*) in cattle grazed areas than those in areas with no cattle exposure. Although *Salmonella* prevalence in manure pit samples was 30% and was consistent with what has been detected in fecal material from swine pens on swine farms (33%; 95% CI, 20–48; [25], the prevalence of *Salmonella* in soil samples from swine farms (6%; 95% CI, 4–8) was lower than what has been previously detected in soil samples from swine farm (23%; 95% CI, 12–37) environments from 18 different farms across five states in the United States [25]. Interestingly, the prevalence of *Salmonella* in soil samples on swine farms was more consistent with what was detected in soil samples from conservation areas in this study (5%; 95% CI, 4–7) and in sand on beaches used for human bathing in the United Kingdom (6%; 95% CI, 3–10; [47], which had no direct livestock exposure.

*Salmonella* prevalence in raccoon fecal and soil samples on farms and conservation areas may not have differed in our study because not only did all of the study sites have some level of human use, but they all were located within the boundaries of the Grand River watershed in Ontario, which is heavily impacted by agriculture and humans [39]. Although manure spreading [48], human refuse [49], or pet dog feces [50] are possible sources of *Salmonella* exposure to raccoons, *Salmonella* occurrence in natural water has been well documented [21, 51–52], and transmission to terrestrial animals may occur through contact with or consumption of contaminated water [53]. In a major produce region of California, *Salmonella* was detected most frequently in water samples (7%; 95% CI, 4–11) compared to samples from soil/sediment (3%; 95% CI, 1–4), wildlife feces/fecal swabs (4.2%; 95% CI, 3–6), beef cattle feces (0.1%; 95% CI, 0–1), and pre-harvest produce (0/271) [54]. Because some of the same *Salmonella* serovars were detected in both wildlife and water samples, it was suggested that *Salmonella* might be transported between wildlife and water [54].

In the Grand River watershed, *Salmonella* was detected in 78% of water samples collected from agricultural/rural and urban tributaries [21]. This is quite high relative to other watersheds in Canada and was more comparable to *Salmonella* levels found in rivers in Georgia, U.S.A, which are heavily affected by agriculture [21]. The profile of *Salmonella* serovars detected from aquatic samples from the Grand River Watershed [21] appeared to be similar to what we detected in raccoon fecal samples in this study. Similarly, in a study of free-ranging urban and rural raccoons in Indiana, Morse et al. [55] found that the *Salmonella* serovars detected in raccoon feces and lymph nodes reflected the profile of *Salmonella* serovars found in water, fish, and mussel samples collected from the environment the raccoons were trapped two years prior. Because raccoons frequently use and feed in aquatic habitats [56] and water can act as a vehicle for *Salmonella* transport [57], raccoons may be exposed to *Salmonella* from contaminated rivers, streams, and tributaries.

Many of the most common serovars found in raccoon fecal samples in this study were also among the most common serovars found in soil samples. The similarity in *Salmonella* serovars found in raccoons feces and their local environment suggests that raccoons are exchanging *Salmonella* with their environment and that raccoons and/or soil may be used as sentinels for environmental *Salmonella*. For example, *Salmonella* Enteritidis, which was the second most common serovar detected in raccoons from Pennsylvania in 2011 [36], was detected infrequently in raccoon fecal samples and soil samples in this study as well as water samples from the Grand River watershed [21]. In addition, *Salmonella* Oranienburg is not historically a common serotype infecting human populations in Ontario; however, in 2010 it was listed as one of the top ten serovars affecting humans in Ontario for the first time in many years [58]. Likewise in this study, Oranienburg was detected commonly in raccoon and environmental samples in 2011 and in 2012, but was not detected at all in 2013.

Some *Salmonella* serovars, such as *Salmonella* Agona and Typhimurium var. DT 104 Copenhagen, may have been detected primarily in raccoon and environmental samples from swine farms due to contamination of the environment with livestock manure. FoodNet Canada found that among *Salmonella* in Ontario livestock in 2010, Agona and Typhimurium var. Copenhagen were among the most common serovars in swine [59]. *Salmonella* Typhimurium var. DT 104 Copenhagen phagetype DT 10 has also been detected in retail pork samples from Ontario [60]. Some *Salmonella* serovars detected in the manure pit samples in this study (e.g., Livingstone and Livingstone var. 14) were not commonly found in swine in Ontario [59], so it is possible that some isolates represented serovars better able to survive and persist in the manure pit environment. Also, three of the five farms had other domestic animal species present on the farm, and although the farms reported the waste of other animals was not present in the sampled manure pits, we cannot definitively rule that out. Although we detected some significant differences in the models for the most common serovars for raccoon fecal samples, caution should be taken when interpreting the results because the effective sample size was small and consequently we were unable to fit multivariable models.

We found a significant interaction between sex and season for the carriage of *Salmonella* in raccoon feces. In previous analyses, we also found significant interactions between sex and season for carriage of *Salmonella* on paws and in feces of raccoons [41] and prevalence and number of *Baylisascaris procyonis* in raccoon feces [35]. Females may have a lower prevalence of *Salmonella* and *Baylisascaris* than males earlier in the season due to behavioral differences associated with females provisioning young and maintaining smaller home-ranges from May to July [61–63]. If female raccoons are not moving around as often or as far as males during provisioning of their young, this might result in them having less exposure to sources of *Salmonella* and other pathogens in their environment. It also has been suggested that behavior may play a role in higher prevalence and transmission of *Salmonella* observed between men and women [64] and male and female wild boars [65].

Both male and female raccoons were more likely to carry *Salmonella* in their feces from August to November than May to July. These differences may be related to seasonal changes in diet [66] similar to the effect of feed composition and structure on survival of *Salmonella* in the gastrointestinal tracts of swine [67]. The higher prevalence of *Salmonella* detected in raccoons from August to November may have been related to the increased prevalence of *Salmonella* found in soil samples during the same time period; however, the intensity of transmission between raccoons and the environment is difficult to determine because the soil could also be contaminated from raccoon or other wildlife feces. Interestingly, *Clostridium difficile* was more likely to occur in raccoon fecal samples from May to July than August to November in the same population of raccoons examined in this study [68], suggesting the epidemiology and microbiology of carriage of these organisms in raccoon feces may be quite different.

Although *Salmonella* occurrence in raccoon fecal samples was negatively associated with rainfall during both seasons, the relationship was more pronounced from August to November than May to July. Higher rainfall was also associated with lower carriage of *Salmonella* in mongoose feces [29], and a negative correlation was found between rainfall the previous 30 days prior to sample collection and *Salmonella* excreted by quokkas (*Setonix brachyurus*), an Australian marsupial [69]. Dietary shifts associated with rainfall have been hypothesized to alter the intestinal microbiota and influence fecal shedding of *Salmonella* in free-ranging kangaroos [43]. It has also been suggested that rainfall might help disseminate *Salmonella* throughout the environment [29]. No associations were detected between *Salmonella* occurrence in soil and rainfall, which suggests raccoons, might be exposed to *Salmonella* through environmental sources other than the soil. Contaminated water is one possible exposure of *Salmonella* to raccoons because during drier periods, water availability is more limited, and animals might aggregate at common water sources where pathogens might be concentrated [29].

*Salmonella* prevalence was higher in soil and manure pit samples later than earlier in the season, which is similar to the findings of other studies [25, 70]. Although *Salmonella* can contaminate soil through animal waste, human wastewater, or contaminated water, *Salmonella* prevalence and survival in the soil depends on a variety of factors including temperature, moisture, soil type, presence of plants, exposure to sunlight (ultraviolet), protozoan predation, and the initial number of organisms present [71]. A combination of these factors might explain the seasonal and yearly variation detected in *Salmonella* prevalence in soil samples in this study. In general, higher temperatures increase mortality of *Salmonella* in soil [72] and decrease survival of *Salmonella* in stored swine manure [73]. There is also a risk of under-estimating the prevalence of *Salmonella* in environmental samples when using culture-based methods because bacterial cells can enter a viable, but non-culturable state [74].

The ability of *Salmonella* to survive in the environment may play an important role in its transmission between host animals, dissemination, and persistence in animal and human populations [21]. In Michigan, the feces of raccoons were found to contribute to as much as 60% of fecal material in urban storm water sewers that drain into streams and rivers [75]. Once raccoons are colonized and shedding the bacteria, they have the potential to disseminate *Salmonella* throughout the environment. Although the duration that raccoons shed the same *Salmonella* serovar in their feces has been reported to be up to 30 days, raccoons have been found to carry different *Salmonella* serovars in their feces each month they were sampled [35]. This further suggests that raccoons are not maintaining long-term colonization of these bacteria without re-exposure from the environment [35].

We detected many *Salmonella* serovars in raccoon feces that were among the most commonly reported in humans from Ontario in 2011–2012, including Typhimurium, Heidelberg, Newport, I 4,[5],12:b:-, Thompson, and Infantis [58, 60]. Raccoons can shed *Salmonella* intermittently [35, 55] and have been found to carry multiple *Salmonella* [55, 76–77], so all of the *Salmonella* serovars in raccoon feces may not have been detected. *Salmonella* prevalence in raccoon feces in this study was 26% (95% CI, 23–28), which is consistent with what has previously been found in rural raccoons living in southwestern Ontario (27%; 95% CI, 12–46; [35]. Although the sensitivity of *Salmonella* testing could be increased by testing whole fecal specimens rather than fecal swabs and by sampling over multiple consecutive days [78], those methods were not used in this study because we were unable to obtain whole fecal samples from the majority of the raccoons captured and raccoons were not trapped and resampled consecutively over multiple days.

Antimicrobial resistance was rarely detected in *Salmonella* isolates from raccoon feces in this study, and the prevalence was similar to what has been previously reported from raccoons from Ontario and Pennsylvania (4%; [35]; 2%; [36]), but lower than what has been reported from rural and urban raccoons from Indiana (16%; 7/43; 95% CI 6.8–30.7; [55]. The observed prevalence was much lower than that reported for swine (62%; 38/61; 95% CI 49.0–74.4) from on-farm surveillance, chicken (47%; 180/382; 95% CI 42.0–52.3) and pork samples from retail meat surveillance (69%, 25/36; 95% CI 51.9–83.7), and humans (26%; 950/3601; 95% CI 24.9–27.9) in Ontario [60]. The source of antimicrobial resistance on swine farms that did not report antimicrobial use and in conservation areas is unclear. A meta-analysis comparing the prevalence of antimicrobial resistance in organic and conventional poultry, swine, and beef concluded that bacterial isolates from conventional production systems exhibited more antimicrobial resistance than isolates from organic production, but that some resistant isolates were still detected from samples from organic farms [79]. Agricultural and surface run-off may play a role in the dissemination of *Salmonella* [21] and resistant bacteria [80], but it is also possible that raccoons and other wildlife such as gulls and waterfowl may acquire *Salmonella* and antimicrobial resistant bacteria from contaminated areas and transport it to more natural areas. Other potential sources of *Salmonella* and antimicrobial resistant bacteria for raccoons include contaminated pet [81, 82] or human food [83] and/or waste [84].

Fluoroquinolones and cephalosporins are the antimicrobials likely to be used in clinical treatment of human salmonellosis [85]; only 2 *Salmonella* isolates were resistant to cephalosporins and none were resistant to fluoroquinolones. Although raccoons have been found to carry many *Salmonella* serovars of public health significance and have the potential to transport them to humans, their role in disseminating resistant *Salmonella* is less clear.

## Conclusion

Our results indicate raccoons living on farms and conservation areas in southwestern Ontario may be important hosts of *Salmonella*. Occurrence of *Salmonella* in raccoons and soil was affected by year and climatic variables, but demographic factors were also important predictors for *Salmonella* occurrence in raccoon feces. Although location type did not affect the frequency of *Salmonella* occurrence in either sample type, indicating *Salmonella* in the environment is widespread, more sites would need to be sampled in order to determine if our results hold true across the entire Grand River Watershed. Different serovars and antimicrobial resistance phenotypes dominated on farms versus conservation sites, suggesting raccoons locally acquire *Salmonella* and that they may be a useful sentinel species for *Salmonella* and associated antimicrobial resistance in the environment. Because many of the most common *Salmonella* serovars causing illness in humans and impacting livestock health were also found in raccoon fecal samples, raccoons may have the potential to transport *Salmonella* to humans, livestock, and the environment regardless of the original source of infection.

## Supporting Information

S1 Appendix Multivariable models. (DOCX)

S1 Table Results from univariable multi-level and exact ^a^ logistic regression models showing associations between the occurrence of *Salmonella* in raccoon fecal, soil, and manure pit samples with respect to raccoon age and sex for raccoon samples, location type, year, season, and climatic variables in Ontario, Canada. (DOCX)

S2 Table Results from univariable multi-level logistic regression models showing associations between the most common *Salmonella* serovars compared to all others found in raccoon samples with respect to raccoon age and sex, location type, year, and season in Ontario, Canada. (DOCX)
