# Supplementary material for: Impact of Season, Demographic and Environmental Factors on Salmonella Occurrence in Raccoons (Procyon lotor) from Swine Farms and Conservation Areas in Southern Ontario
Source: PLoS One. 2016 Sep 9;11(9):e0161497. doi: 10.1371/journal.pone.0161497 (PMC5017689; doi:10.1371/journal.pone.0161497)
Supplement: S1 Appendix — (DOCX) [file pone.0161497.s001.docx]

**S1 Appendix. Multivariable models.**

Before proceeding with the multivariable model construction, we examined the correlation between mean temperature 30 days prior to sample collection and sum of rainfall over 30 days prior to sample collection using Pearson’s correlation coefficient. If the terms were moderately correlated,│ρ │> 0.8, then the two variables were modelled separately to avoid collinearity.

In creating final models, main effects that were not significant were removed from the multivariable main effects model one at a time, and then interactions considered to be biologically plausible were included in the model individually to assess their significance. For outcomes concerning raccoon fecal samples, all possible interactions between location type, season, and year were tested in addition to the interactions between age and sex and mean temperature and sum of rainfall. For outcomes concerning soil samples, all possible interactions between location type, year, and season were tested as well as the interaction between mean temperature and sum of rainfall.

Contrasts were constructed using the lincom command to estimate odds ratios, 95% confidence intervals (CIs), and p-values based on model-derived coefficients to examine interactions between categorical variables in the final multi-level models. For significant interactions involving continuous and categorical variables, the relationships between the variables and the outcome were visualized based on graphing predicted means.

For outcomes concerning raccoon fecal, soil, and manure pit samples, multivariable models were fitted. A main effects model was constructed for manure pit samples, but no interactions were tested due to a small effective sample size. Due to small effective sample sizes, multivariable models of the most common serovars were not constructed. A multivariable model for dumpster samples was not created because dumpster samples were collected during only one year.
