# Supplementary material for: Impact of Season, Demographic and Environmental Factors on Salmonella Occurrence in Raccoons (Procyon lotor) from Swine Farms and Conservation Areas in Southern Ontario
Source: PLoS One. 2016 Sep 9;11(9):e0161497. doi: 10.1371/journal.pone.0161497 (PMC5017689; doi:10.1371/journal.pone.0161497)
Supplement: S1 Table — (DOCX) [file pone.0161497.s002.docx]

**S1 Table. Results from univariable multi-level and exact ^a^ logistic regression models showing associations between the occurrence of *Salmonella* in raccoon fecal, soil, and manure pit samples with respect to raccoon age and sex for raccoon samples, location type, year, season, and climatic variables in Ontario, Canada.**

| **Predictor** | **Sub-Category** | **Univariable models for *Salmonella* according to sample type** | | | | | | | | | | | |
| --- | --- | --- | --- | --- | --- | --- | --- | --- | --- | --- | --- | --- | --- |
|  |  | Raccoon feces ^b,^ ^c^ | | | Variance [VPC] | | | Soil ^b,^ ^d^ | | | Manure pit ^b^ | | |
|  |  |  | | | (95% CI) | | |  | | |  | | |
|  |  | Odds | 95% CI | *P* | Site- | Animal- | Sample-- | Odds | 95% CI | *P* | Odds | 95% CI | *P* |
|  |  | Ratio |  |  | level | level | level | Ratio ^d^ |  |  | Ratio |  |  |
| Sex | Female | REF |  |  |  |  |  | — | — | — | — | — | — |
|  | Male | **1.48** | **1.05–2.09** | **0.025** | 0.13 [3.1] | 0.79 [18.8] | [78.1] | — | — | — | — | — | — |
|  |  |  |  |  | (0.03–0.61) | (0.33–1.91) |  |  |  |  |  |  |  |
| Age | Adult | REF |  |  |  |  |  | — | — | — | — | — | — |
|  | Juvenile | 1.34 | 0.93–1.93 | 0.117 | 0.09 [2.1] | 0.81 [19.3] | [78.5] | — | — | — | — | — | — |
|  |  |  |  |  | (0.01–0.58) | (0.34–1.94) |  |  |  |  |  |  |  |
| Location type | Conservation Area | REF |  |  |  |  |  | REF |  |  | — | — | — |
|  | Swine farm | 0.76 | 0.44–1.31 | 0.322 | 0.10 [2.4] | 0.79 [18.9] | [78.7] | 1.07 | 0.70–1.64 | 0.757 | — | — | — |
|  |  |  |  |  | (0.02–0.57) | (0.33–1.92) |  |  |  |  |  |  |  |
| Season | May to July | REF |  |  |  |  |  | REF |  |  | REF |  |  |
|  | Aug. to Nov. | **1.45** | **1.06–1.99** | **0.022** | **0.10 [2.4]** | **0.81 [19.3]** | **[78.3]** | **2.61** | **1.59–4.31** | **< 0.001** | 3.15 | 0.911–12.8 | 0.063 |
|  |  |  |  |  | **(0.02–0.57)** | **(0.33–1.95)** |  |  |  |  |  |  |  |
| Year ^e^ | 2012 (2011 REF) | 1.01 | 0.69–1.50 | 0.951 | 0.12 [2.9] | 0.74 [17.8] | [79.2] | 1.15 | 0.72–1.84 | 0.562 | 0.42 | 0.10–1.63 | 0.223 |
|  |  |  |  |  | (0.02–0.62) | (0.02–0.62) |  |  |  |  |  |  |  |
|  | 2013 (2011 REF) | 0.66 | 0.43–1.02 | 0.060 | 0.12 [2.9] | 0.74 [17.8] | [79.2] | **0.45** | **0.24–0.82** | **0.010** | **0.08** | **0.07**–**0.45** | **0.001** |
|  |  |  |  |  | (0.02–0.62) | (0.30–1.86) |  |  |  |  |  |  |  |
| Sum of rainfall | Over 30 days prior | **1.00** | **0.99–1.00** | **0.003** | 0.14 [3.3] | 0.80 [18.9] | [77.8] | 1.00 | 0.99–1.00 | 0.332 | 1.00 | 0.99–1.01 | 0.747 |
|  |  |  |  |  | (0.03–0.65) | (0.33–1.93) |  |  |  |  |  |  |  |
| Mean temperature | Over 30 days prior | 1.01 | 0.96–1.06 | 0.636 | 0.12 [2.8] | 0.79 [18.8] | [78.3] | **0.57** | **0.37–0.87** | **0.009** | 1.02 | 0.88–1.18 | 0.830 |
|  |  |  |  |  | (0.02–0.60) | (0.32–1.91) |  |  |  |  |  |  |  |
| Mean temperature squared | Over 30 days prior | — | — | — |  |  |  | **1.02** | **1.00–1.03** | **0.008** | — | — | — |
|  |  |  |  |  |  | | |  |  |  |  |  |  |

^a^ Exact logistic regression was used to model manure pit samples.

^b^ Significant differences are in bold, the dash indicates “Not Applicable”, REF = referent group, and CI = confidence interval.

^c^ Random effects included site and animal.

^d^ Random effect for site was not included in the model because it did not improve model fit based on AIC and BIC; it explained only a small amount of the variance (8.4 x 10^-31^ to 1.4 x 10^-28^), and its removal had little to no impact on the coefficients in the model.

^e^ Wald’s χ^2^ test for year was *P* = 0.090 for raccoon fecal samples and *P* =0.009 for soil samples. For manure pit samples, the results for 2013 (2012 REF) were (OR = 0.18; 95% CI = 0.02-1.07; *P* = 0.0374).
