# Supplementary material for: Impact of Season, Demographic and Environmental Factors on Salmonella Occurrence in Raccoons (Procyon lotor) from Swine Farms and Conservation Areas in Southern Ontario
Source: PLoS One. 2016 Sep 9;11(9):e0161497. doi: 10.1371/journal.pone.0161497 (PMC5017689; doi:10.1371/journal.pone.0161497)
Supplement: S2 Table — (DOCX) [file pone.0161497.s003.docx]

|  |  | **Univariable models for most common *Salmonella* serovars compared to all others found in raccoon fecal samples ^a^** | | | | | | | | | | | | | | | | | |
| --- | --- | --- | --- | --- | --- | --- | --- | --- | --- | --- | --- | --- | --- | --- | --- | --- | --- | --- | --- |
| Predictor | Sub-category | Newport | | Variance [VPC] | | | Oranienburg ^b^ | | Variance [VPC] | | I:4,[5],12:b:- | | Variance [VPC] | | | Thompson ^b^ | | Variance [VPC] | |
|  |  | (*n* = 279) | | (95% CI) | | | (*n* = 279) | | (95% CI) | | (*n* = 279) | | (95% CI) | | | (*n* = 279) | | (95% CI) | |
|  |  | OR | *P* | Site- | Animal- | Sample- | OR | *P* | Site- | Sample- | OR | *P* | Site- | Animal | Sample | OR | *P* | Site- | Sample- |
|  |  | (95% CI) |  | level | level | level | (95% CI) |  | Level | level | (95% CI) |  | level | level | level | (95% CI) |  | level | level |
| Location | Conservation |  |  |  |  |  |  |  |  |  |  |  |  |  |  |  |  |  |  |
| Type | Area (REF) |  |  |  |  |  |  |  |  |  |  |  |  |  |  |  |  |  |  |
|  | **Swine Farm** | **6.94** | **0.042** | **1.39 [26.2]** | **0.62 [11.7]** | **[62.1]** | 0.06 | 0.277 | 14.24 [81.2] | [18.8] | 0.14 | 0.386 | 8.51 [63.6] | 1.57 [11.7] | [24.6] | 0.19 | 0.074 | 1.10 [25.1] | [74.9] |
|  |  | **(1.07–45.09)** |  | **(0.28–6.83)** | **(0.02–15.71)** |  | (0.00–9.39) |  | (1.93–104.93) |  | (0.002–12.0) |  | (1.29–56.23) | (0.15–16.72) |  | (0.03–1.18) |  | (0.26–4.57) |  |
| Sex | Female (REF) |  |  |  |  |  |  |  |  |  |  |  |  |  |  |  |  |  |  |
|  | **Male** | 0.94 | 0.857 | 2.46 [38.8] | 0.59 [9.3] | [51.9] | **0.41** | **0.039** | **20.87 [86.4]** | [13.6] | 0.91 | 0.862 | 8.58 [64.0] | 1.53 [11.4] | [24.6] | 1.09 | 0.836 | 1.52 [31.6] | [68.4] |
|  |  | (0.46–1.91) |  | (0.57**–**10.64) | (0.02**–**17.00) |  | **(0.17–0.96)** |  | **(2.80–155.53)** |  | (0.31–2.68) |  | (1.26–58.62) | (0.14–16.67) |  | (0.49–2.41) |  | (0.38–6.18) |  |
| Season | May to July |  |  |  |  |  |  |  |  |  |  |  |  |  |  |  |  |  |  |
|  | (REF) |  |  |  |  |  |  |  |  |  |  |  |  |  |  |  |  |  |  |
|  | Aug. to Nov. | 1.69 | 0.169 | 2.46 [38.9] | 0.61 [9.6] | [51.7] | 0.91 | 0.825 | 20.06 [85.9] | [14.1] | 0.67 | 0.510 | 9.30 [65.9] | 1.53 [10.8] | [23.3] | 1.77 | 0.172 | 1.60 [32.7] | [67.3] |
|  |  | (0.80–3.59) |  | (0.57**–**10.59) | (0.02**–**14.60) |  | (0.40–2.07) |  | (2.68–150.12) |  | (0.21–2.18) |  | (1.35–64.13) | (0.15–16.05) |  | (0.78–4.03) |  | (0.40–6.43) |  |
| Age | Adult (REF) |  |  |  |  |  |  |  |  |  |  |  |  |  |  |  |  |  |  |
|  | **Juvenile** | 0.89 | 0.765 | 2.48 [38.8] | 0.63 [9.8] | [51.4] | **0.35** | **0.020** | **20.80 [86.3]** | [13.7] | 0.64 | 0.405 | 8.54 [65.8] | 1.14 [8.8] | [25.4] | 1.66 | 0.213 | 1.46 [30.7] | [69.3] |
|  |  | (0.41–1.92) |  | (0.57**–**10.75) | (0.02**–**15.69) |  | **(0.14–0.85)** |  | **(2.75–157.33)** |  | (0.22–1.85) |  | (1.27–57.53) | (0.07–18.63) |  | (0.75–3.71) |  | (0.36–5.93) |  |
| Year ^e^ | **2012** | 0.85 | 0.710 | 2.97 [44.5] | 0.41 [6.2] | [49.3] | **3.56** | **0.011** | **25.15 [88.4]** | [11.6] | 0.37 | 0.219 | 7.40 [58.0] | 2.07 [16.2] | [25.8] | 0.52 | 0.177 | 1.38 [29.6] | [70.4] |
|  | **(2011 REF)** | (0.35–2.05) |  | (0.71**–**12.35) | (0.01**–**36.42) |  | **(1.34–9.42)** |  | **(3.29–192.51)** |  | (0.08–1.80) |  | (1.04–52.70) | (0.20–21.69) |  | (0.20–1.34) |  | (0.33–5.68) |  |
|  | 2013 | 2.38 | 0.078 | 2.97 [44.5] | 0.41 [6.2] | [49.3] | Omitted ^d^ | — | — | — | 0.54 | 0.404 | 7.40 [58.0] | 2.07 [16.2] | [25.8] | 0.54 | 0.254 | 1.38 [29.6] | [70.4] |
|  | (2011 REF) | (0.91–6.24) |  | (0.71**–**12.35) | (0.01**–**36.42) |  |  |  |  |  | (0.12–2.32) |  | (1.04–52.70) | (0.20–21.69) |  | (0.19–1.55) |  | (0.33–5.68) |  |

**S2 Table. Results from univariable multi-level logistic regression models showing associations between the most common *Salmonella* serovars compared to all others found in raccoon samples with respect to raccoon age and sex, location type, year, and season in Ontario, Canada.**

^a^ Random effects included site and animal. Significant differences are in bold. *n* = sample size, REF = referent group, OR = odds ratio, CI = confidence interval.

^b^ Random effect for animal was not included because it did not improve model fit based on AIC and BIC; it explained only a small amount of the variance (4.0 x 10^-39^ – 0.15), and its removal had

little to no impact on the coefficients in the model.

^c^ Wald’s χ^2^ test for year was *P* = 0.092 for Newport, *P* = 0.428 for I:4,[5],12:b:-, and *P* = 0.324 for Thompson.

^d^  All observations for 2013 (*n* = 61) were omitted from the analysis for year for Oranienburg due to computational problems of no positive observations in that group.
